# Acute generalized exanthematous pustulosis from imatinib

**DOI:** 10.1186/1710-1492-10-S2-A9

**Published:** 2014-12-18

**Authors:** Lisa W Fu, Amanda Jagdis, Jason K Lee

**Affiliations:** 1 Department of Medicine, McGill University, Montreal, QC, Canada; 2 Division of Clinical Immunology and Allergy, Department of Medicine, University of Toronto, Toronto, ON, Canada

## Background

Acute generalized exanthematous pustulosis (AGEP) is rare cutaneous drug reaction involving drug-specific T lymphocytes and neutrophilic inflammation. Imatinib is a tyrosine kinase inhibitor used widely in treatment of chronic myeloid leukemia (CML). Rash is a common side effect of imatinib, occurring in up to 30% of patients. However, there are only two case reports to date describing AGEP secondary to imatinib.

## Case presentation

A 71-year-old woman known for chronic myeloid leukemia presented with acute onset of generalized pruritus, accompanied by small erythematous papules, and diffuse general edema affecting the face, and upper and lower extremities. The rash developed over a few hours and was associated with shortness of breath, chills, and dysuria. The rash persisted for 14 days at which point a 5 day course of prednisone was prescribed which led to improvement. Imatinib (generic) had been started 3 months earlier; was temporarily interrupted for 2-3 weeks after 1 month; then restarted 2 months prior to onset of the rash. On exam there was pitting edema to lower 2/3 anterior tibia, erythematous papular generalized excoriated rash with xerosis. No blistering, sloughing of skin, target lesions, or pustules. CBC normal, creatinine unremarkable and urine negative for eosinophils.

Imatinib (generic) was discontinued and brand name Gleevec ® was started. Two weeks later the rash recurred. Eosinophil count increased to 2.84 x 10^9^/L. A skin biopsy was arranged which showed parakeratosis containing neutrophils with formation of intracorneal pustules, in keeping with AGEP.

Gleevec ® was discontinued and Nilotinib 400mg PO BID was substituted 2 weeks later. The rash resolved and eosinophilia decreased over the next month and has not recurred.

## Conclusion

AGEP is a rare complication of imatinib therapy, and should be considered in patients who present with atypical rash on imatinib.

## Consent

Written informed consent was obtained from the patient for publication of this abstract and any accompanying images. A copy of the written consent is available for review by the Editor of this journal.

